# Benchmark cases for a multi-component Lattice–Boltzmann method in hydrostatic conditions

**DOI:** 10.1016/j.mex.2020.101090

**Published:** 2020-10-09

**Authors:** E.P. Montellà, B. Chareyre, S. Salager, A. Gens

**Affiliations:** a University Grenoble Alpes (UGA), CNRS, Grenoble INP, 3SR, Grenoble F-38000, France; b Department of Civil and Environmental Engineering. Universitat Politècnica de Catalunya - CIMNE, Barcelona, Spain

**Keywords:** Capillarity, Pore scale, Simulation, Two-phase flow, Lattice Boltzman

## Abstract

Hydrostatic properties of partially saturated granular materials at the pore scale are evaluated by the lattice Boltzmann method (LBM) using Palabos implementation of the multi-component multiphase Shan-Chen model. Benchmark cases are presented to quantify the discretization errors and the sensitivity to geometrical and physical properties. This work offers practical guidelines to design LBM simulations of multiphase problems in porous media. Namely, a solid walls retraction procedure is proposed to reduce discretization errors significantly, leading to quadratic convergence. On this basis the equilibrium shapes of pendular bridges simulated numerically are in good agreement with the Young-Laplace equation. Likewise, entry capillary pressure and meniscus profiles in tubes of various cross-sectional shapes are in agreement with analytical predictions. The main points of this article are summarized as:

• Benchmark cases for a multi-component Lattice-Boltzmann method are illustrated to be a guideline to calibrate the method in hydrostatic conditions.
• A wall retraction procedure is introduced to minimize discretization errors.

## Graphical abstract

**Figure:**
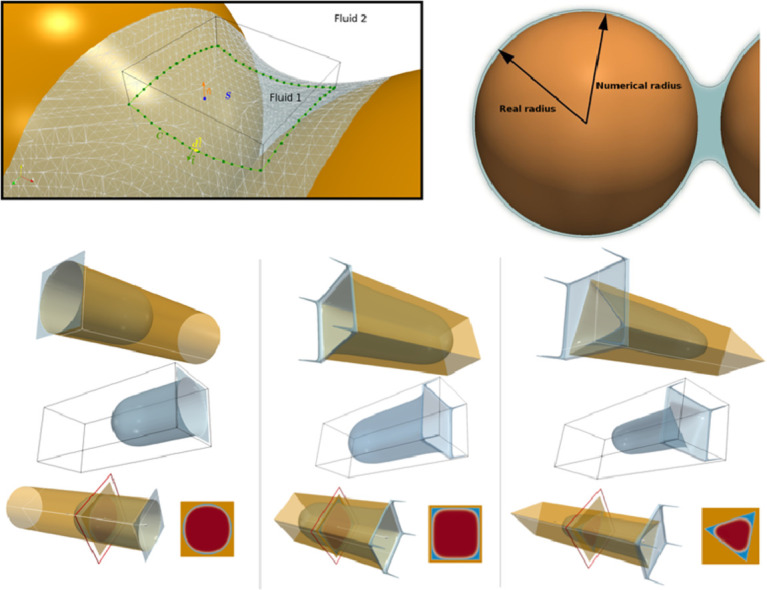

Specifications Table

**Table:** 

Subject Area:	Engineering
More specific subject area:	Soil mechanics – Partially saturated media
Method name:	– Benchmark cases for a multi-component Lattice-Boltzmann method inhydrostatic conditions – Wall retraction procedure
Name and reference of original method:	– The multicomponent Shan and Chen method Lattice Boltzmann method: [1] X. Shan, H. Chen, Lattice boltzmann model for simulating flows with multiple phases and components, Physical Review E 47 (3) (1993) 1815. [2] X. Shan, H. Chen, Simulation of nonideal gases and liquid-gas phase transitions by the lattice boltzmann equation, Physical Review E 49 (4) (1994) 2941 – The Mayer and Stowe-Princen (MS-P) model [3] R. P. Mayer, R. A. Stowe, Mercury porosimetrybreakthrough pressure forpenetration between packed spheres, Journal of colloid Science 20 (8) (1965) 893–911.
Resource availability:	Palabos open source library

## Introduction

Fully resolved numerical solutions to multiphase pore-scale problems are used increasingly in simulation domains extracted from 3D imaging. There is, simultaneously, a growing interest in the development of simplified methods based on pore-network idealizations since simulating spatio-temporal evolutions at the REV scale would require tremendous computational resources when intricate couplings are at play. This was done primarily for low-porosity materials (typically rock materials) [1], [2]. Extensions of the pore network approach to granular media appeared more recently and they still rely on strong assumptions and simplifications [3], [4], [5]. In the pore-network approach the movements of phases and interfaces are governed by local rules such as the entry capillary pressure, the capillary pressure – saturation curve and the capillary forces. When the local capillary pressure is larger than the entry capillary pressure of a pore throat the non-wetting phase penetrates it invading the pore body. Several approaches can be considered to compute the entry capillary pressure. The most common approximations are the Haines incircle method and the Mayer–Stowe–Princen(MS-P) method [3], [4], [5]. Unfortunately, these approximations predict just a single pressure value missing crucial information before and after the invasion that could be provided with an accurate local capillary pressure - saturation relationship. Establishing those local rules is another use-case for fully resolved solutions – for elementary microstructures in that case. The lattice Boltzmann method (LBM) is frequently used for producing well resolved solutions. In this study we assess the accuracy of a multiphase LBM scheme for the solution to hydrostatic problems. A background motivation of this work is the extension of pore-network methods to deformable granular media, following the strategy employed previously for saturated flow [6]. We therefore focus on elementary microstructures. Nevertheless the conclusions in terms accuracy and mesh dependency apply equally well to simulations of REVs. It is, thus, worth mentioning that this benchmark is intended to serve as validation of the numerical simulation method to be applied in a practical situation. More specifically, this benchmark is used to justify the mesh resolution and flow conditions employed in [7], where the pore space is decomposed into small subsets of three spheres (pore throats) that are solved independently to determine the main hydrostatic properties.

The LBM is a mesoscopic model capable of simulating fluid dynamics in complex geometries [8]. Many works using the LBM have focus on a single saturating fluid phase and proven to be successful [9], [10], [11]. However, multiphase LB models in partial saturation have less satisfactory results due to the complexity of phases interactions. Several multiphase LB models have been proposed in the literature: the color model [12], the pseudopotential (Shan-Chen) model [13], [14] or the free-energy model [15]. The so-called Shan-Chen model has single- and multi-component variants which both apply to the problem of immiscible phases. The single-component method is simpler. It has been used to simulate, for instance, flow in porous media with realistic rock geometries [16], [17] or the hysteretic response of idealized sphere-pack systems in drainage-imbibition [18]. More recently, [19] investigated with this method the meniscus profile and the effect of contact angle on fluid displacement through polygonal capillary tubes. According to [20] however, the gas-liquid interfaces tend to be more diffused in single component simulations, which may hinder the approach of strongly immiscible situations. Fewer studies have applied the multicomponent method [21], [22] although it is supposed to reflect the fluid composition more accurately [23]. Very few authors - if any - examined the accuracy of the multicomponent scheme for hydrostatic solutions. In this paper, the multicomponent Shan and Chen model is employed using the open-source lattice Boltzmann library Palabos [24] to complement the results and conclusions of previous studies and benchmarks [23], [25].

The paper is organized as follows: in section *Numerical method*, the lattice Boltzmann method and the Shan-Chen model are briefly described; section *Model calibration* explains the way that surface tension and contact angle can be computed and tuned; in the section *Validation* LBM results are compared to analytical solutions for capillary tubes and pendular bridges between spheres; finally, conclusions are drawn in last section.

## Numerical method

### Lattice Boltzmann method

In this section we provide a brief explanation of the LB method. The LBM has its origin in the lattice gas automata (LGA) [26], a kinetic model based on discrete space-time field. While LGA method described the evolution of individual particles on a lattice, the LBM solves a discrete kinetic equation (Boltzmanns equation) for a particle distribution function $f^{\sigma }\left(x,t\right)$. Where the superscript σindicates the fluid component, xrefers to the lattice node and tis the time. In the LBM, the motion of fluid is described by the lattice Boltzmann equation. Based on the simple and popular Bhatnagar–Gross–Krook (BGK) collision operator [27], the standard LB equation can be expressed as follows:

(1) $$f_{k}^{\sigma }\left(x_{k}+e_{k}\Delta t,t+\Delta t\right)-f_{k}^{\sigma }\left(x_{k},t\right)=\frac{-\Delta t}{\tau^{\sigma }}\left(f_{k}^{\sigma }\left(x_{k},t\right)-f_{k}^{\sigma ,eq}\left(x_{k},t\right)\right)$$

where $\tau^{\sigma }$is the rate of relaxation towards local equilibrium, $f_{k}^{\sigma ,eq}$is the equilibrium distribution function, Δtis the time increment, $e_{k}$are the discrete velocities which depend on the particular velocity model, in this work, D3Q19 (three-dimensional space and 19 velocities) model is used, and kvaries from 0 to Q−1representing the directions in the lattice. The left-hand side of Eq. (1)describes the streaming step (particles move to the nearest node following its velocity direction) whereas the right-hand side stands for the collision operator (particles arriving to the nearest node modify their velocity towards a local equilibrium). The collision operator correspond to the viscous term in the Navier–Stokes equation. For the D3Q19 model, the discrete velocity set $e_{k}$is written as:

(2) $$e_{k}=\left\{\begin{array}{c} \left(0,0,0\right)\\ \left(\pm 1,0,0),\;(0,\pm 1,0),\;(0,0,\pm 1\right)\\ \left(\pm 1,\pm 1,\pm 1\right) \end{array}\right\}\;w_{k}=\left\{\begin{array}{c} 1/3\\ 1/18\\ 1/36 \end{array}\right\}\;\begin{array}{c} k=0\\ k=1,...,6\\ k=7,...,18 \end{array}$$

where $w_{k}$are the weight factors.

The local equilibrium $f_{k}^{\sigma ,eq}$depends on the lattice type and the macroscopic variables $\rho^{\sigma }=\sum_{k}f_{k}^{\sigma }$(density) and $\rho^{\sigma }u^{\sigma }=\sum_{k}f_{k}^{\sigma }e_{k}$(momentum) [28]. The equilibrium distribution can be seen as an expansion of the Maxwell–Boltzmanns distribution function for low Mach numbers:

(3) $$f_{k}^{\sigma ,eq}=\rho^{\sigma }w_{k}\left\{1+\frac{1}{c_{s}^{2}}\left(e_{k}\cdot u^{\sigma ,\mathrm{eq}}\right)-\frac{1}{2c_{s}^{2}}\left(u^{\sigma ,\mathrm{eq}}\cdot u^{\sigma ,\mathrm{eq}}\right)+\frac{1}{2c_{s}^{4}}{\left(e_{k}\cdot u^{\sigma ,\mathrm{eq}}\right)}^{2}\right\}$$

where $c_{s}=\frac{1}{\sqrt{3}}$is the speed of sound and $u^{\sigma ,\mathrm{eq}}$is the equilibrium velocity defined as [13], [14]:

(4) $$u^{\sigma ,\mathrm{eq}}=u^{{}^{\prime }}+\frac{\tau^{\sigma }F_{\sigma }}{\rho^{\sigma }}$$

where $u^{{}^{\prime }}=\frac{\sum_{\sigma }\frac{\rho^{\sigma }u^{\sigma }}{\tau^{\sigma }}}{\sum_{\sigma }\frac{\rho^{\sigma }}{\tau^{\sigma }}}$is an effective velocity and $F_{\sigma }$is the total force (including body forces and the fluid–fluid interactions that will be presented in *Pseudopotential model*section) acting on each component.

### Pseudopotential model

The interactions between components (or phases) in the Shan and Chen model are defined by pairwise interaction forces. These forces modify the collision operator through an equilibrium velocity and produce a repulsive effect between the phases. We focus on biphasic mixtures (i.e., σ= 1,2), described two distributions $f_{k}^{\sigma }\left(x,t\right)$. Hereafter, $\rho_{w}$and $\rho_{nw}$will refer to the wetting and non-wetting phases. $\rho_{o}$is defined as the reference density which is kept at $\rho_{o}=1$. The non-local force responsible for the fluid-fluid interaction is expressed as:

(5) $$F_{\sigma }\left(x\right)=-\Psi \left(x\right)\sum_{\bar{\sigma }}G_{\sigma \bar{\sigma }}\sum_{k}\Psi_{k}\left(x+e_{k}\right)e_{k}$$

where $\Psi_{k}$is the interparticle potential that induces phase separation and $G_{\sigma \bar{\sigma }}$is the interaction strength between components σ,$\bar{\sigma }$.

Previous works [14], [29], [30], [31] have employed several interparticle potentials. For simplicity, we consider $\Psi_{k}=\rho_{k}$, as done in other papers [18]. The interactions within each component, $G_{11}$and $G_{22},$are set equal to zero for biphasic mixtures. On the other hand, the interactions between components, $G_{12}$= $G_{21},$are set positive in order to induce a repulsive force between the phases. Low values of $G_{12}$lead to dissolution processes seen in typical miscible mixtures. On the contrary, significantly high values of $G_{12}$result into almost immiscibile binary mixtures with sharp interface prone to numerical instability. Thus, special attention must be paid when choosing the interaction strength as it controls the surface tension and immiscibility of the mixture. The interaction force given by Eq. (5)leads to a non-spherical pressure tensor $\bar{\bar{P}}$deduced from the condition: $-\nabla \bar{\bar{P}}+\nabla \bar{\bar{P_{o}}}=F_{\sigma }\left(x\right)+F_{\bar{\sigma }}\left(x\right)$, where $\bar{\bar{P_{o}}}=\bar{\bar{I}}c_{s}^{2}\left(\rho_{\sigma }+\rho_{\bar{\sigma }}\right)$is the ideal pressure tensor [32], [33]. The components of the pressure tensor can be computed as:

(6) $$P_{ij}\left(x\right)=c_{s}^{2}\left[\rho_{\sigma }\left(x\right)+\rho_{\bar{\sigma }}\left(x\right)\right]I_{ij}+\frac{G}{2}\Psi^{\sigma }\sum_{k=0}^{N-1}w_{k}\Psi^{\bar{\sigma }}\left(x+c_{k}\right)c_{ki}c_{kj}+\frac{G}{2}\Psi^{\bar{\sigma }}\sum_{k=0}^{N-1}w_{k}\Psi^{\sigma }\left(x+c_{k}\right)c_{ki}c_{kj}$$

Following Eq. (6), the non-ideal equation of state (EOS) can be determined as:

(7) $$p=c_{s}^{2}\sum_{\sigma }\rho_{\sigma }+c_{s}^{2}\sum_{\sigma \bar{\sigma }}G_{\sigma \bar{\sigma }}\Psi_{\sigma }\Psi_{\bar{\sigma }}$$

## Model calibration

### Contact angle

**Fig. 1:**
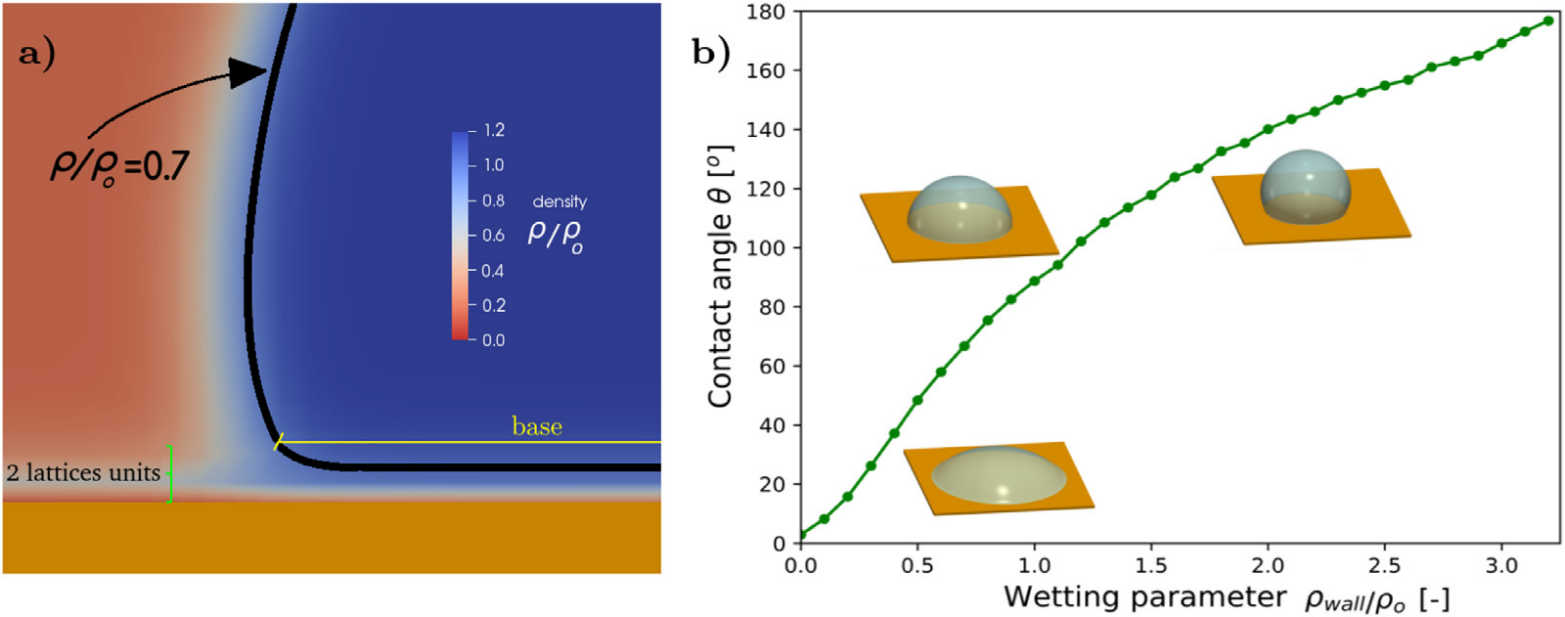
(a): Detail of the fluid-fluid-solid phase transitions in a droplet test. The interface between the non-wetting fluid (blue) and the wetting fluid (red) is defined by the contour line $\rho_{w}/\rho_{o}=0.7$(black dark line). The bottom part of the image is the solid wall (orange). The interface forms a contact angle of approximately $102^{\circ }$for a $\rho_{wall}/\rho_{o}=1.2$at a reference point situated 2 lattice units above the solid wall (green marker). (b): contact angle versus pseudo density $\rho_{wall}$of the solid wall.

The fluid-solid interaction is implemented in the Shan-Chen model by a mid-grid bounce back scheme applied on the boundaries [34]. This scheme assigns fluid properties to the solid wall. Among them, the pseudo wall density $\rho_{wall}$(non-real density assigned to the nodes of the solid boundary) controls wettability [19], [35], [36]. The interparticle potential at the wall in Eq. (5)is $\Psi =\rho_{wall}$). We perform simulations of static droplets on a flat solid surface and we analyze the dependence of $\rho_{wall}$on the contact angle. Simulations are performed in a 150×150×150 lattice domain. Once the simulation is stable and converged, the base length (b) and the height (h) are measured. Knowing the geometrical characteristics of the droplet allows us to determine the contact angle $\frac{\theta }{2}=tan^{-1}\left(\frac{2h}{b}\right)$[37](see Fig. 1(b)). Some error is introduced during the base measurement due to the thickness of the interface layer in the vicinity of the solid wall. In order to overcome the problem, the base and height of the droplet are determined from a reference point located 2 lattice units away from the wall (Fig. 1(a)). Moreover, as further discussed in *Numerical method* section, $\rho_{w}/\rho_{o}=0.7$is the density threshold used for positioning the interface (dark line in Fig. 1(a)).

### Surface tension

**Fig. 2:**
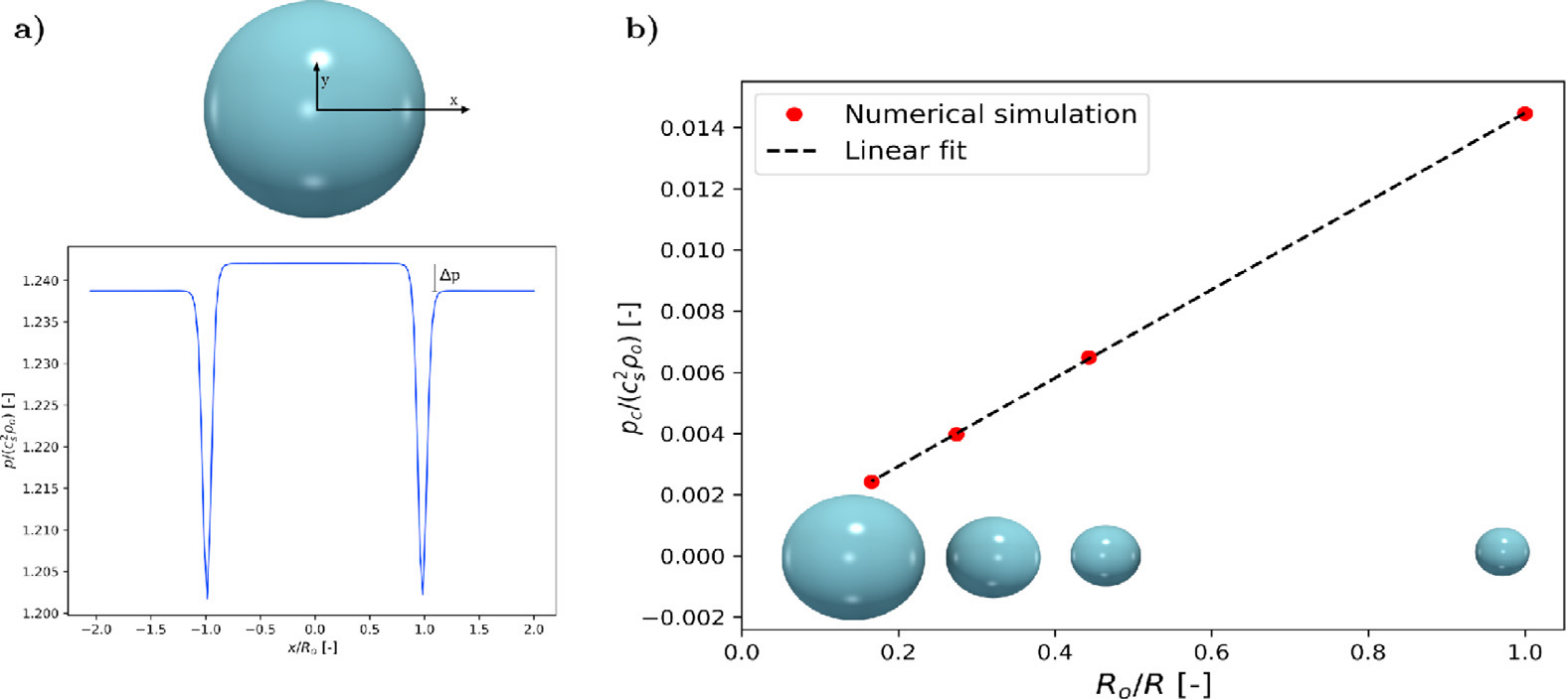
(a): Pressure along a line crossing a spherical droplet (*x*-axis). (b): the evolution of (normalized) capillary pressure with droplet size, the slope of this line defines surface tension.

**Fig. 3:**
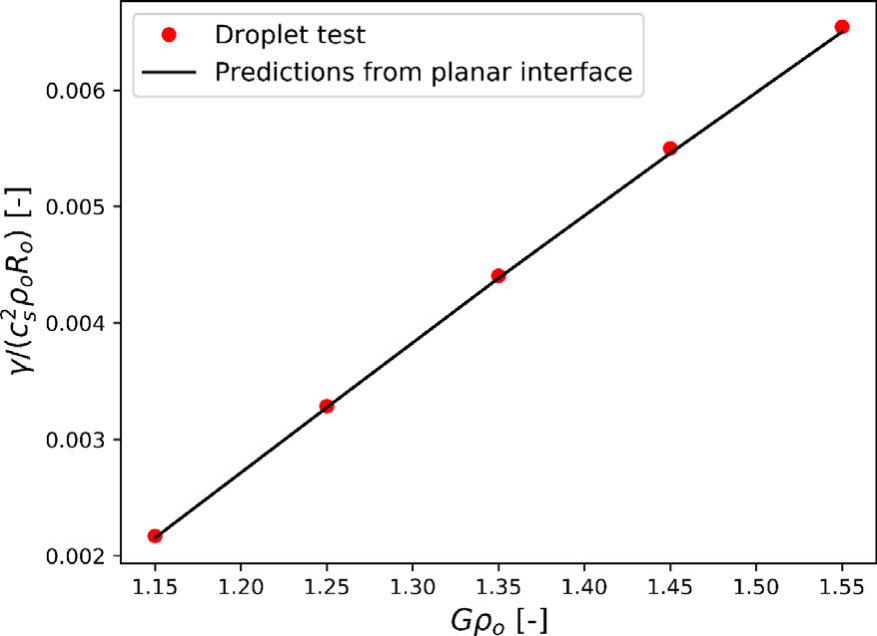
Dependency of surface tension on the interaction strength G. The black line represents the integral of Eq. (10)across a flat interface, the red dots correspond to the droplet test.

Surface tension is adjusted by tuning the interaction between different fluid species. The typical numerical set-up to investigate the surface tension consist of a series of spherical drops with different radii inside a domain with periodic boundary conditions. The droplet and the surrounding fluid are at rest and the pressure difference inside and outside the droplet is balanced by the surface tension according to the Young–Laplace law ($p_{c}=\frac{2\gamma }{R}$). Fig. 2(a) depicts the pressure along a line passing through the center of the droplet. There are two significant drops in pressure when the line crosses the interface [38], it denotes to surface tension. The pressure difference Δpcorresponds to capillary pressure. Fig. 2(b) shows the variation of $p_{c}$versus 1/Rin dimensionless terms ($R_{o}$is the radius of the smallest droplet), where the linear relationship is evidenced. The slope of the linear fit is the interfacial tension $\gamma^{*},$which is determined as $\gamma^{*}=\frac{\gamma }{\rho_{o}c_{s}^{2}R_{o}}$for $G\rho_{o}=1.25$. Different surface tension values are assessed for different interaction strength parameters G(see Fig. 3).

Surface tension can be also be determined based on a two-phase system with a flat interface having a constant pressure in both phases far from the interface [39]. This technique has been adopted in many works relying on the single-component Shan–Chen model [14], [32], [38], [40]. Literature on the multicomponent model is more scarce yet the flat interface has also been used in that case [35]. We reproduced it for comparison with the droplet test. The logic of the analysis is as follows. The pressure inside the bulk phases corresponds to the scalar quantity p. However, near the interface, due to the surface tension contribution, the pressure is defined as a tensor incorporating different pressure components. Moreover, in order to ensure the mechanical stability, the gradient of the pressure tensor must be zero everywhere in the fluid [41]. The symmetry of the surface requires that pis a diagonal tensor $p(x)=p_{xx}e_{x}⊗e_{x}+p_{yy}e_{y}⊗e_{y}+p_{zz}e_{z}⊗e_{z}$with $p_{xx}\left(x\right)=p_{zz}\left(x\right),$where xand zcorrespond to horizontal directions parallel to the flat interface, yrefers to the axis orthogonal to the planar interface and $e_{j}$is a unit vector in the j-direction. Furthermore, $p_{xx}$and $p_{zz}$are function of yonly, while $p_{yy}$is a constant:

(8) $$p_{xx}\left(y\right)=p_{zz}\left(y\right)=p_{T}\left(y\right)$$

(9) $$p_{yy}\left(y\right)=p_{N}\left(y\right)=p\;$$

where $p_{T}$and $p_{N}$are the transverse and normal components of the pressure. Both $p_{T}$and $p_{N}$can be computed using Eq. (6).

Surface tension is obtained by integrating the difference between $p_{T}$and $p_{N}$along a line crossing the interface [39]:

(10) $$\gamma =\int_{-\infty }^{\infty }\left(P_{N}-P_{T}\right)dy=\int_{-\infty }^{\infty }\left(p-p_{T}\left(y\right)\right)dy$$

The results from droplet test and the flat interface test are compared in Fig. 3, they are in good agreement.

### Note on interface thickness

**Fig. 4:**
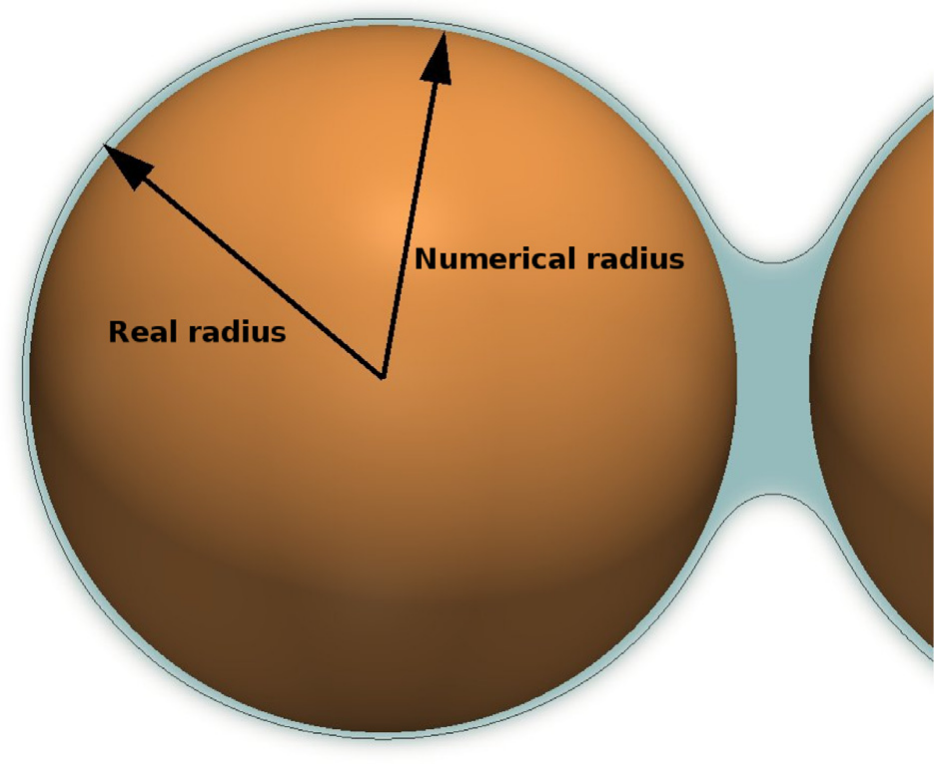
Outline of the wall retraction method. In the LBM mesh the solid boundary is retracted by 2luwith respect to its real position, such that the fluid-fluid contour $\rho_{w}/\rho_{o}=0.7$is nearly coincident with the physical boundary.

The numerical thickness of the interfaces, as seen in Fig. 1 is often considered an issue in the multicomponent Shan-Chen model. Physically inter-molecular interactions lead to a fluid-fluid interface thickness, i.e. a region where the two phases coexist even though they are considered immiscible from a macroscopic point of view. On this basis the fact that the multicomponent Shan Chen model produces diffused interfaces is not strictly unphysical (see Fig. 1(a)). In many applications however the real interface thickness is well below all characteristic lengths of the problem (such as pore size or radius of curvature), hence negligibly small, and then the interface is considered a single surface. In LBM however the thickness of the simulated interface does not correspond to the physical thickness in general. Previous works [42], [43] have evidenced that a fluid-fluid interface of 4–6 lu is required for numerical stability, which could be neglected only at the price of extreme mesh refinement and tremendous computational effort. Some works [23], [25] have attempted to increase the accuracy at fluid-solid interface by introducing new boundary models. Despite the efforts and the better results obtained near the solid region, numerical artifacts are still found to decrease the global accuracy. In order to overcome this issue we propose to redefine the solid boundaries based on a wall retraction logic, including a part of the fluid-solid interface in the region normally occupied by the solid phase in the physical problem (as shown in Fig. 4). This is tested in the next section in the context of capillary tubes.

## Validation

Simple numerical simulations are performed and compared with analytical solutions in order to validate the model. Detailed results are presented for quasi-static displacement of interfaces inside cylindrical tubes and fluid bridges between two spherical bodies.

### Invasion of capillary tubes

**Fig. 5:**
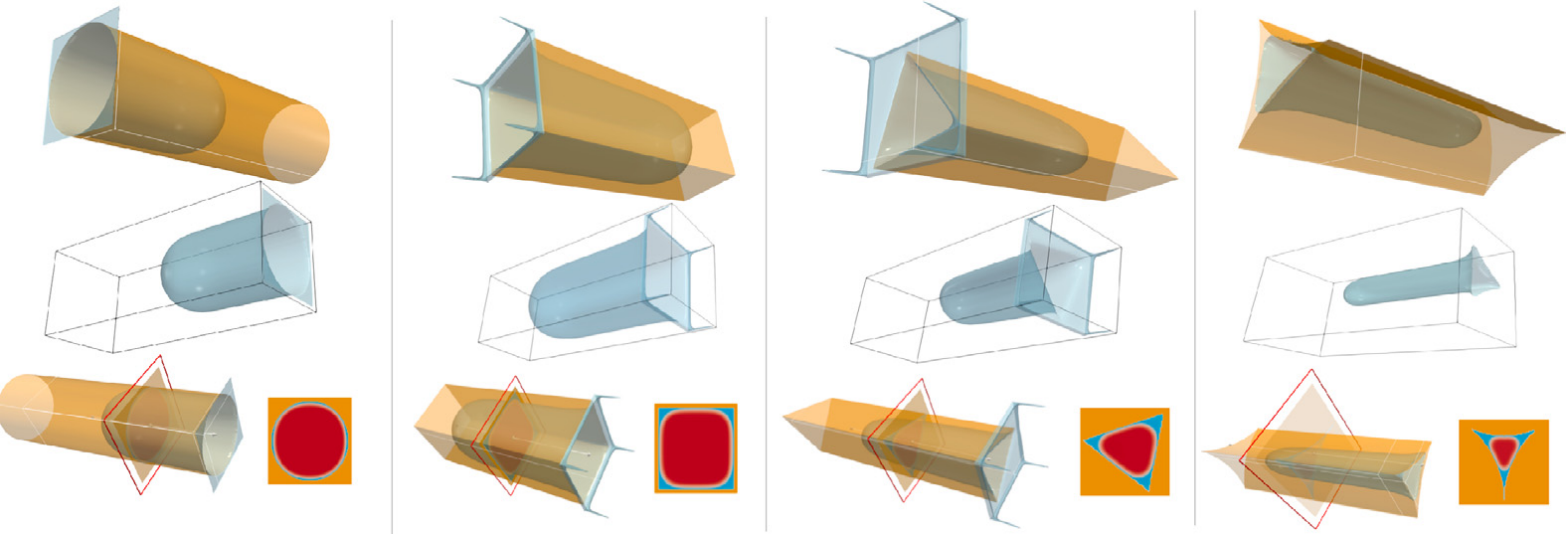
Geometry of the simulated capillary tubes. From left to right: circular, square, triangle and curved boundaries cylinders. On the top row the cylindrical solid walls are displayed translucent to show the interface shape. The middle row illustrates the meniscus shape inside the cylinder. The solid walls are removed for clarity. The bottom row shows the phase distribution in each cylindrical cross-section. The wetting phase (displayed in blue) is retained differently in the corners depending on the cross-sectional shape.

In order to gain better understanding of multiphase flow at the pore scale, it is common to idealize the pores throats as cylindrical capillary tubes [44]. Immiscible flow in such capillary tubes has been simulated with various cross-sectional shapes (Fig. 5). The dimensionless capillary pressure $p_{c}^{*}=\frac{p_{c}L_{c}}{\gamma }$is defined with reference to the following characteristics lengths: $L_{c}$is the radius for the circular cross-section, the side length for the square, the distance between two vertices for the triangle and curved triangle. The fluid displacement corresponds to drainage (invasion by the *nw*-phase) and it is imposed by including mass sink terms in the time integration: wetting phase density is decreased while non-wetting phase density is increased [18]. In order to keep the flow quasistatic the density is only modified when its fluctuation on one time iteration, at interface nodes, is less than a fixed tolerance ($Tol<\frac{|\rho_{it}-\rho_{it+1}|}{\rho^{o}}$). Otherwise the solution is considered out-of-equilibrium and the mass sink is delayed.

#### MS-P method

The Mayer and Stowe-Princen (MS-P) model predicts the capillary pressure and the curvature of the arc meniscus of a fluid droplet of infinite length inside a cylindrical tube [45], [46], [47]. The assumptions of the MS-P method are that that capillary pressure is uniform and that there is no longitudinal curvature away from the main terminal meniscus. Under these assumptions the cross-sectional radius of curvature R(see Fig. 11) defines the total curvature and, after Young-Laplace equation,

(11) $$p_{c}=\gamma /R$$

Furthermore, the balance of forces at equilibrium implies a relationship between capillary pressure and surface tension. The force due to the pressure difference on the cross-sectional area must balance the force from surface tension at the interfaces. Thus,

(12) $$p_{c}A_{nw}=\gamma \left(P_{s}cos\theta +P_{ns}\right)$$

where $P_{s}$is the length of the line between the non-wetting phase and the solid, $P_{ns}$is the perimeter of the interface between the wetting phase and the non-wetting phase, and $A_{nw}$is the area filled with the non-wetting phase. The MS-P method consist in deducing Rby combining Eqs. (11)and (12):

(13) $$R=\frac{A_{nw}}{P_{s}cos\theta +P_{ns}}$$

From now on the MS-P is considered exact for cylindrical throats and used as a reference for comparisons. The errors in LBM solutions will be evaluated using two possible approaches:

(14) $$Error_{p}=\frac{p^{MSP}-p_{e}^{LBM}}{p^{MSP}}$$

where $p_{e}^{LBM}$is the entry pressure obtained in the saturation curves (Fig. 7).

(15) $$Error_{k}=\frac{k^{MSP}-k^{LBM}}{k^{MSP}}$$

where $k^{MSP}$is the curvature defined by the MS-P (the inverse of the radius of Eq. (13)) compared with the curvature of the main meniscus after achieving the entry pressure. $k^{LBM}$is defined in Appendix A.

**Fig. 6:**
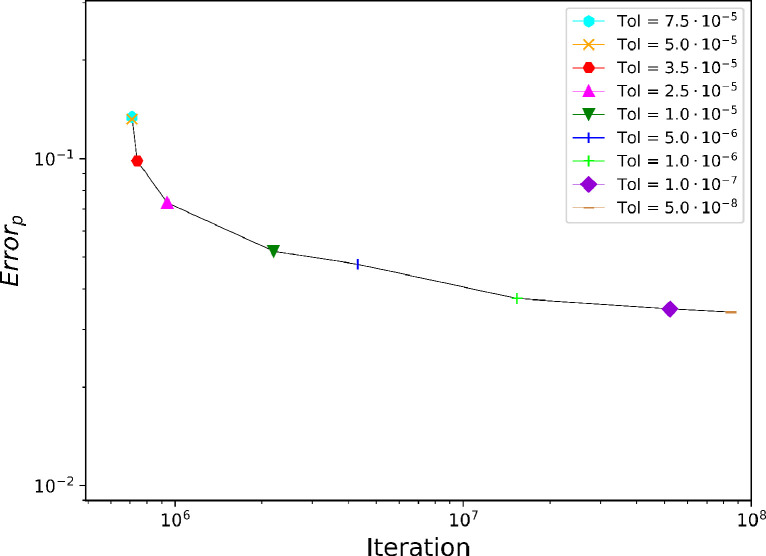
Entry capillary pressure predicted by LBM and total number of time iterations for different values of the tolerance. The error starts to increase significantly from $Tol=10^{-5}\%$

**Fig. 7:**
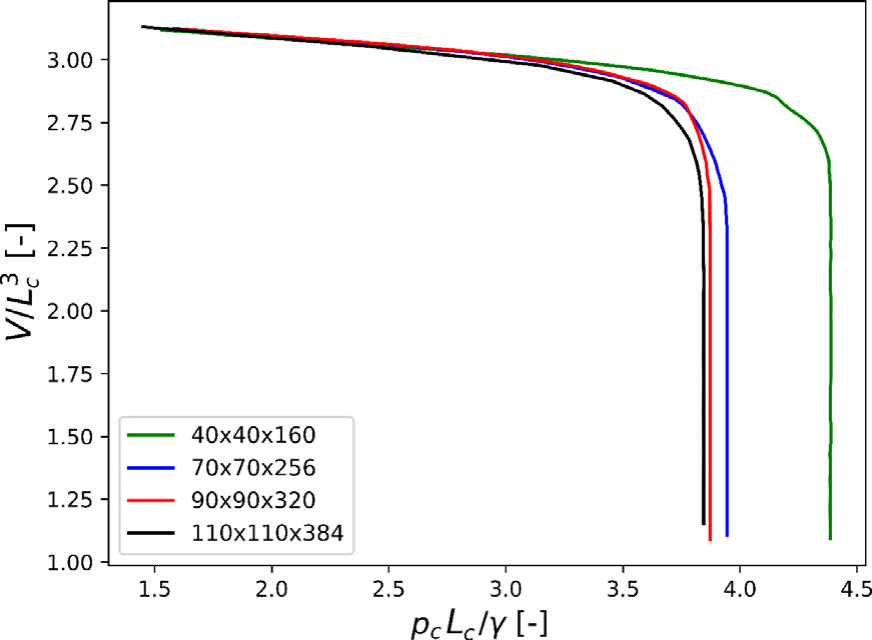
Primary drainage of square-shaped capillary tubes with different discretizations.

#### Results

The entry capillary pressure $p_{e}^{LBM}$in the LBM simulations is deduced from drainage curves similar to the plots in Fig. 7, where Vis the volume occupied by the wetting phase within the tube. The dimensionless capillary pressure increases until the nw-phase breaks through, it then reaches a stationary value of $p_{c}$which corresponds to the entry pressure $p_{e}^{LBM}$. The drainage of the circular tube has been repeated with different values of tolerance (Tolmentioned above) to quantify the perturbation by dynamic effects. The total number of iterations and the difference between $p_{e}^{LBM}$and the MS-P prediction for the different tolerance values are plotted on Fig. 6. Note that the difference is not expected to vanish even with very small tolerance since geometrical discretization errors adds to the error relatively independently of dynamic effects. In the sequel of this study we set the tolerance value to $10^{-5},$as it leads to marginal dynamic errors.

**Fig. 8:**
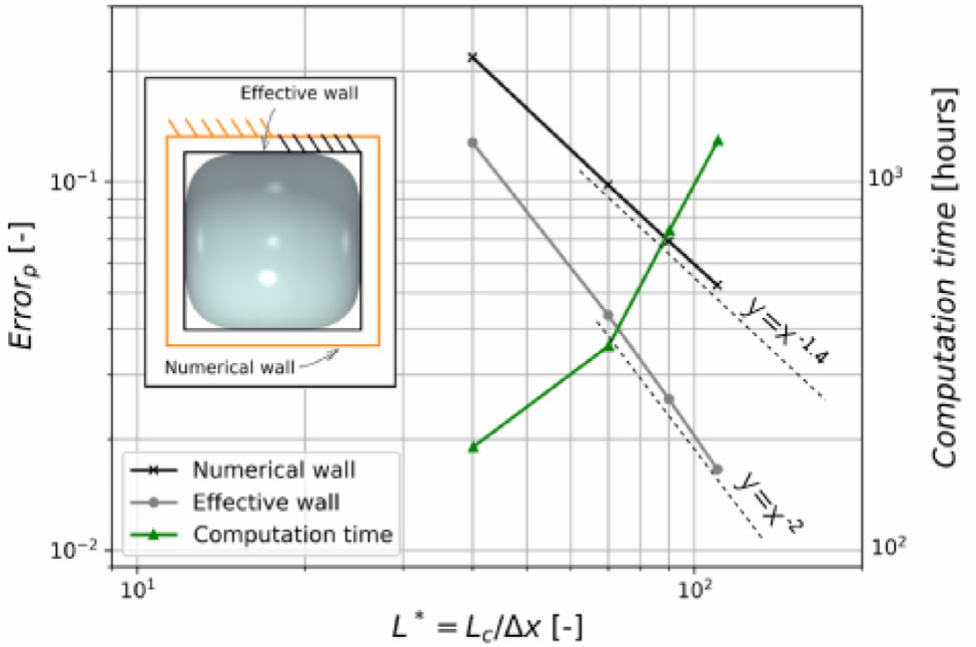
Convergence of the LBM result with mesh refinement, with regard to error defined in Eq. (15). Each simulation is ran in parallel using 8 cores. $L_{c}$is defined as the distance between the numerical walls (unchanged by wall retraction).

Several mesh discretizations have been tested: 40×40×160, 70×70×256, 90×90×320 and 110×110×384 (last value along the axis of the tube). From now on they are referred to as $L_{c}=40lu,$$L_{c}=70lu,$$L_{c}=90lu,$and $L_{c}=110lu,$respectively. The pressure-volume evolution for each mesh size are compared in Fig. 7(for the square-shaped tube). The errors with respect to the MS-P prediction are given by Fig. 8. When the numerical solid wall coincides with the physical wall (no wall retraction) the convergence is superlinear, with an exponent of approximately 1.4. When the interpretation includes wall retraction by two lattice units, the error is smaller and the convergence becomes quadratic, which is a substantial improvement. This technique was used systematically for all simulations presented in the next sections.

**Fig. 9:**
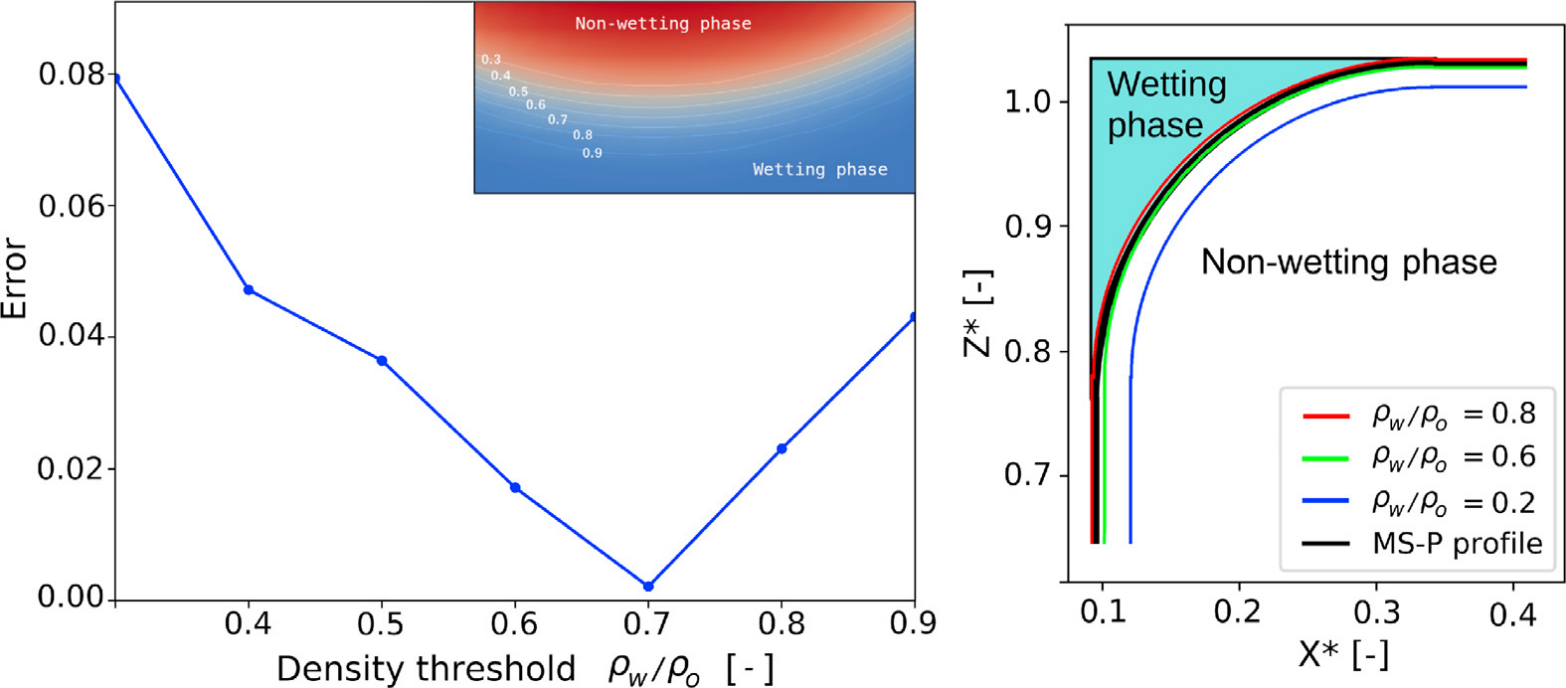
Difference in capillary pressure between LBM theoretical value deduced from meniscus geometry, as a function of the $\rho_{w}$contour selected to define the interface. Sub-figure on the upper-right corner shows details of the density contours. On the right, interface profiles for different $\rho_{w}$are superimposed. Both results correspond to a square cylinder.

A justification of the optimal retraction length is possible by selecting different iso-density surfaces in the result to represent the interface. A consistent definition of the interface should satisfy Eq. (12). Selecting a value of $\rho_{w}/\rho_{o}$to define the interface enables the determination of the geometrical parameter $A_{nw},$$P_{s},$and $P_{ns}$in that equation. The optimal contour is the one which minimizes the deviation from Eq. (12). Based on Fig. 9the optimum is $\rho_{w}/\rho_{o}=0.7,$which corresponds approximately to the average density between both phases. In our results this specific value of density was generally reached approximately two nodes away from the solid nodes, which led to the decision to retract the walls by two lattice units. This value is only valid for $G\rho_{o}=1.25$. Different interaction strength parameters (i.e. other surface tensions) would result in thicker or thinner interfaces, in such case, the same procedure should be repeated to determine the position of the new retraction wall.

**Fig. 10:**
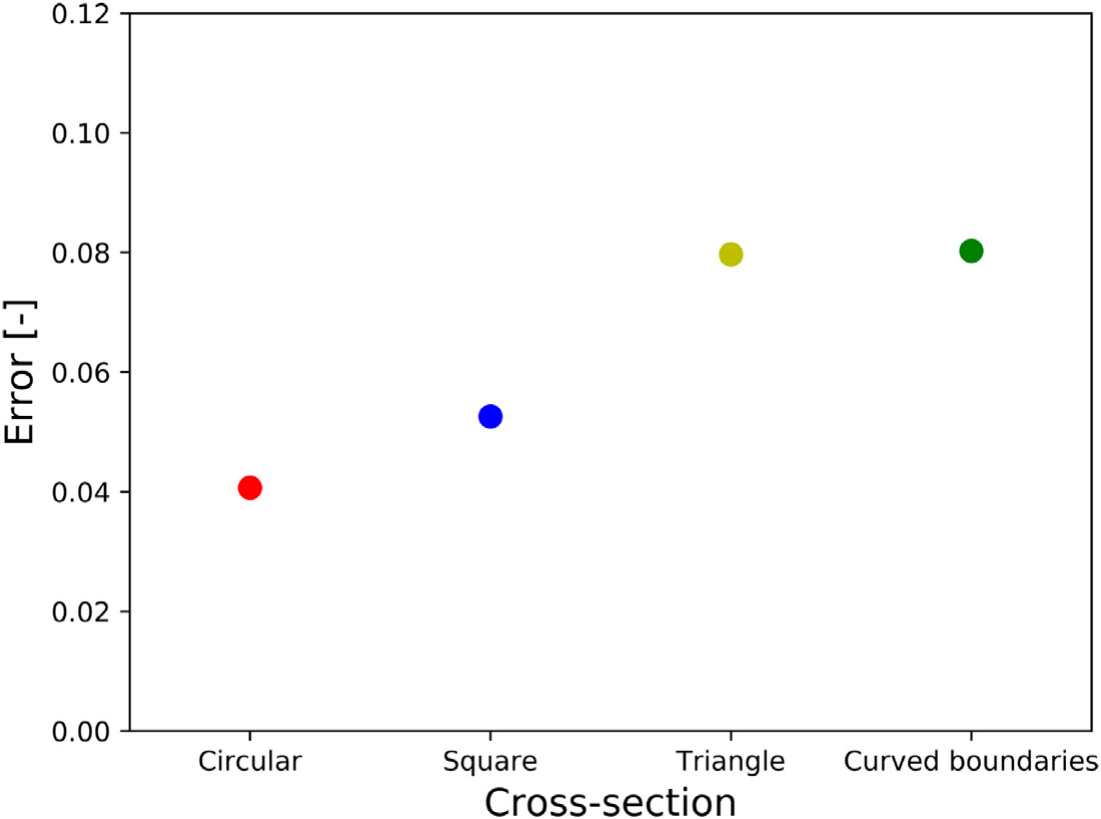
Deviation of LBM results from MS-P for the different cross-sectional shapes for $L_{c}=80$.

**Fig. 11:**
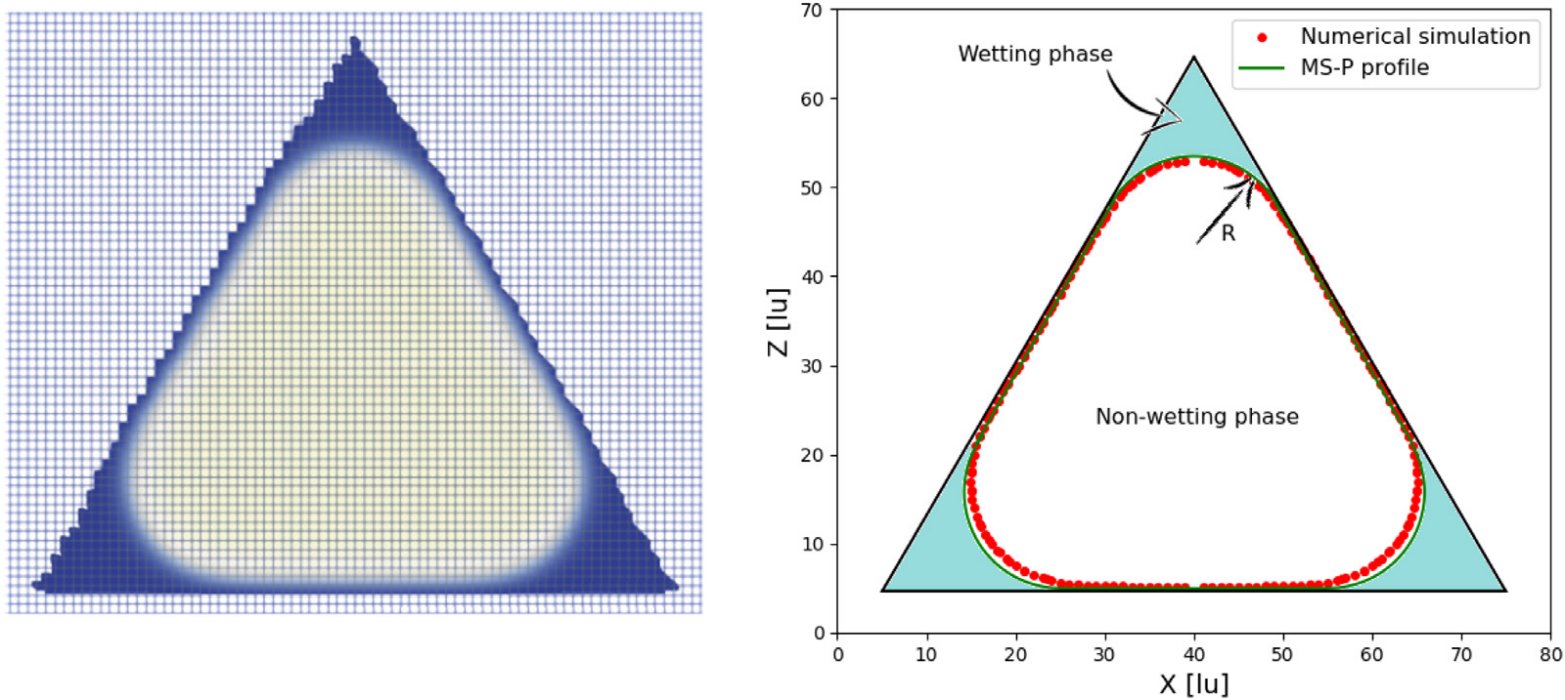
Staircased walls causing non-symmetry of the LBM solution (unequal filling of the corners).

The various cross-sectional shapes have been simulated with domain sizes 80×80×256 $lu^{3}$. The results are compared to the MS-P solution in Fig. 10. We find a reasonable agreement between the simulations and the analytical solution overall. However, larger errors are observed for triangular and curved cross-sections. This can be partly attributed to the artificial roughness introduced by the staircased surfaces. These cross-sections are not aligned to the regular lattice grid. Furthermore, due to the bounce-back boundary condition, these cases lead to mesh-dependent results. In fact, an asymmetry is evidenced in Fig. 11, where the remaining liquid retained in the corners of the equilateral triangle is different in some parts. Nonetheless, Fig. 11shows relatively similar numerical and analytical profiles.

**Fig. 12:**
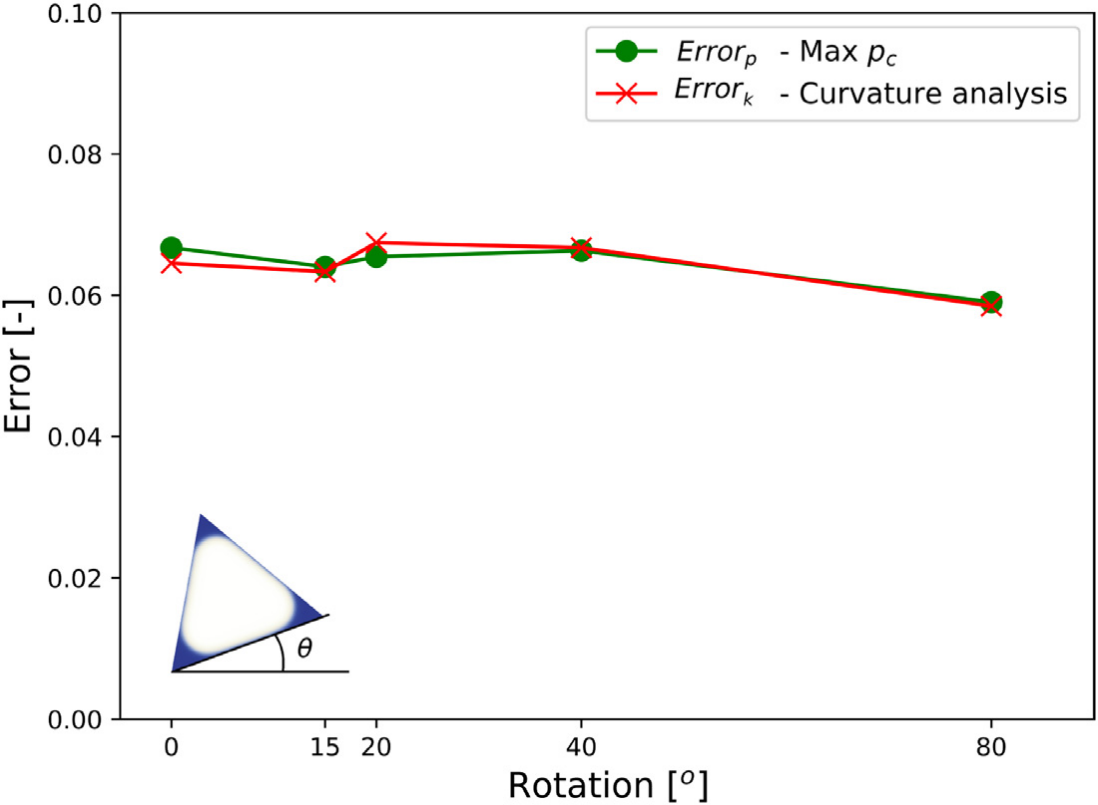
Error on pressure and curvature versus orientation of the throat (relative to LBM grid). $p_{c}^{*}=\frac{p_{c}L}{\gamma }$.

This mesh dependency is frame dependent: it depends on the orientation of the throat with respect to the axis of the grid. The evolution of the errors with rotation is shown in Fig. 12, which reveals that the frame-dependent effects are actually small (of the order of 1%, dominated by other errors).

**Fig. 13:**
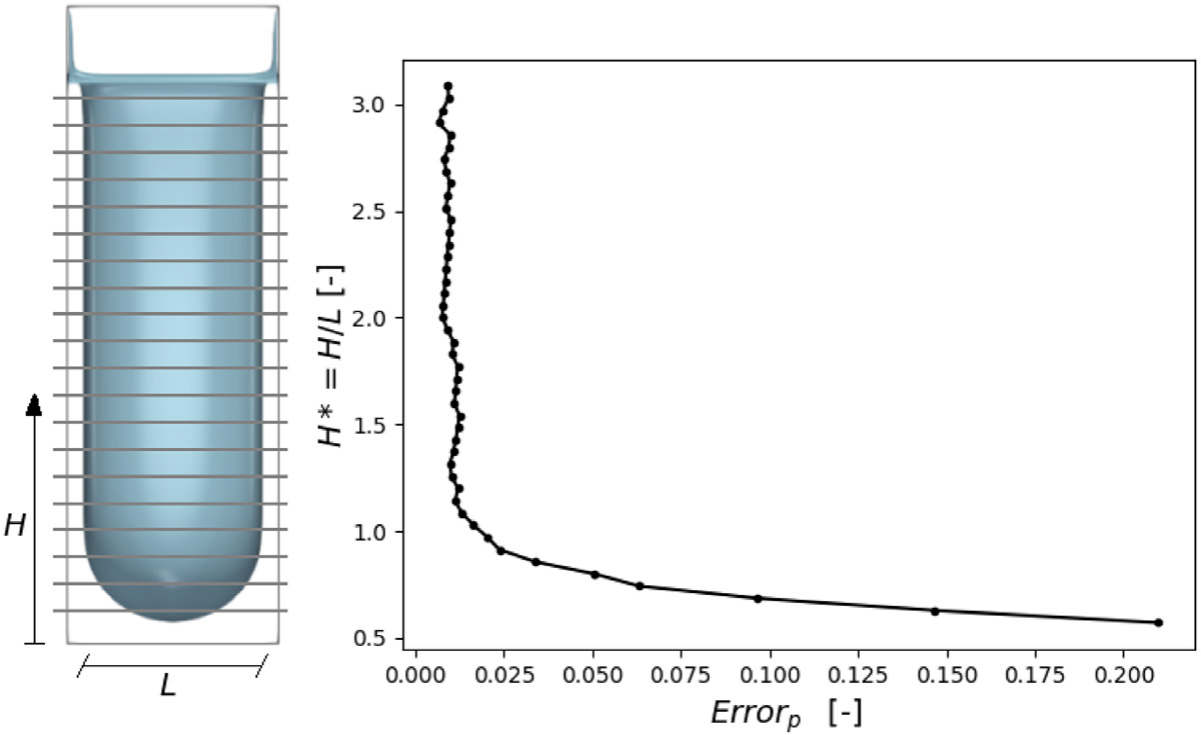
Evolution of the error on pressure by applying MS-P versus distance from the main meniscus.

To conclude this section, we review the hypothesis stating that MS-P solution is valid for cylinders of infinite extension. Due to computation limitations, short domains had to be considered. In order to test the accuracy of the numerical results under these conditions, the error on pressure has been plotted along the cylinder. In other words, capillary pressure was computed using Eq. (12) for various positions of the cross-section in the final, nearly fully invaded, configuration. On the left part of Fig. 13 we observe that the remaining fluid in the corners is parallel to the cylinder walls (no longitudinal curvature). It is concluded that H/L>1is sufficient to approach the situation assumed for the MS-P method, i.e. the cross-section must be behind the main meniscus by a distance approximately equivalent to the throat aperture.

### Pendular bridge

The shape and volume of a pendular bridge between two spheres have been obtained from the LBM and compared to the theoretical solution for a range of capillary pressure.

The simulation setup was as follows: a droplet of the wetting phase was inserted between two identical spheres of radius Rwith a gap equal to 0.14×R. Once a stable state was reached the volume of the liquid bridge was reduced slowly, by an imposed mass sink, until $p_{c}^{*}=\frac{p_{c}R}{\gamma }=0.3$. The shape of the pendular bridge when $p_{c}^{*}=0.3$is compared to the direct solution of Young–Laplace equation [48]in Fig. 15. They show strong similarity. After reaching $p_{c}^{*}=0.3$the LBM simulation was continued by further reducing the amount of wetting phase and recording the volume of the simulated bridge for quantitative comparison with Young–Laplace solution. This was continued until breakage of the bridge.

**Fig. 14:**
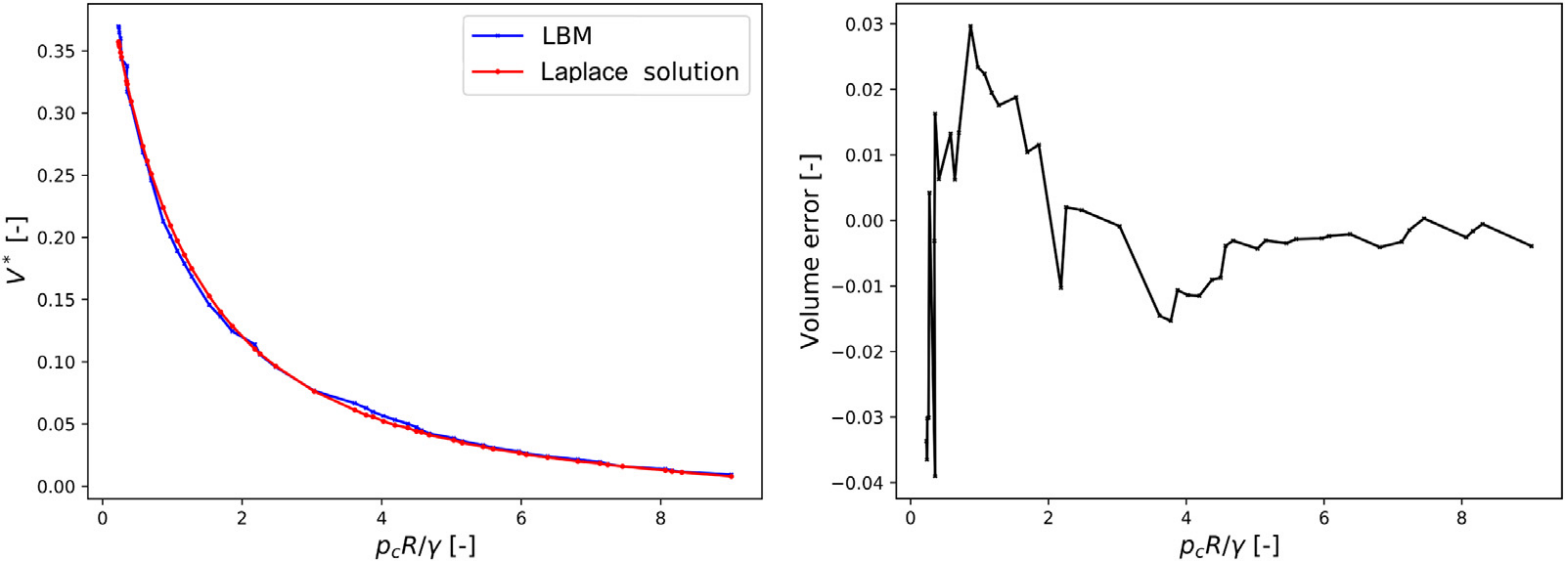
(Left) volume versus capillary pressure for a pendular bridge from LBM and from the numerical solution of Laplace-Young equation. The relative error (right) is the difference between the simulated volume and the theoretical volume normalized by the initial volume $V(p_{c}^{*}=0.3)$.

**Fig. 15:**
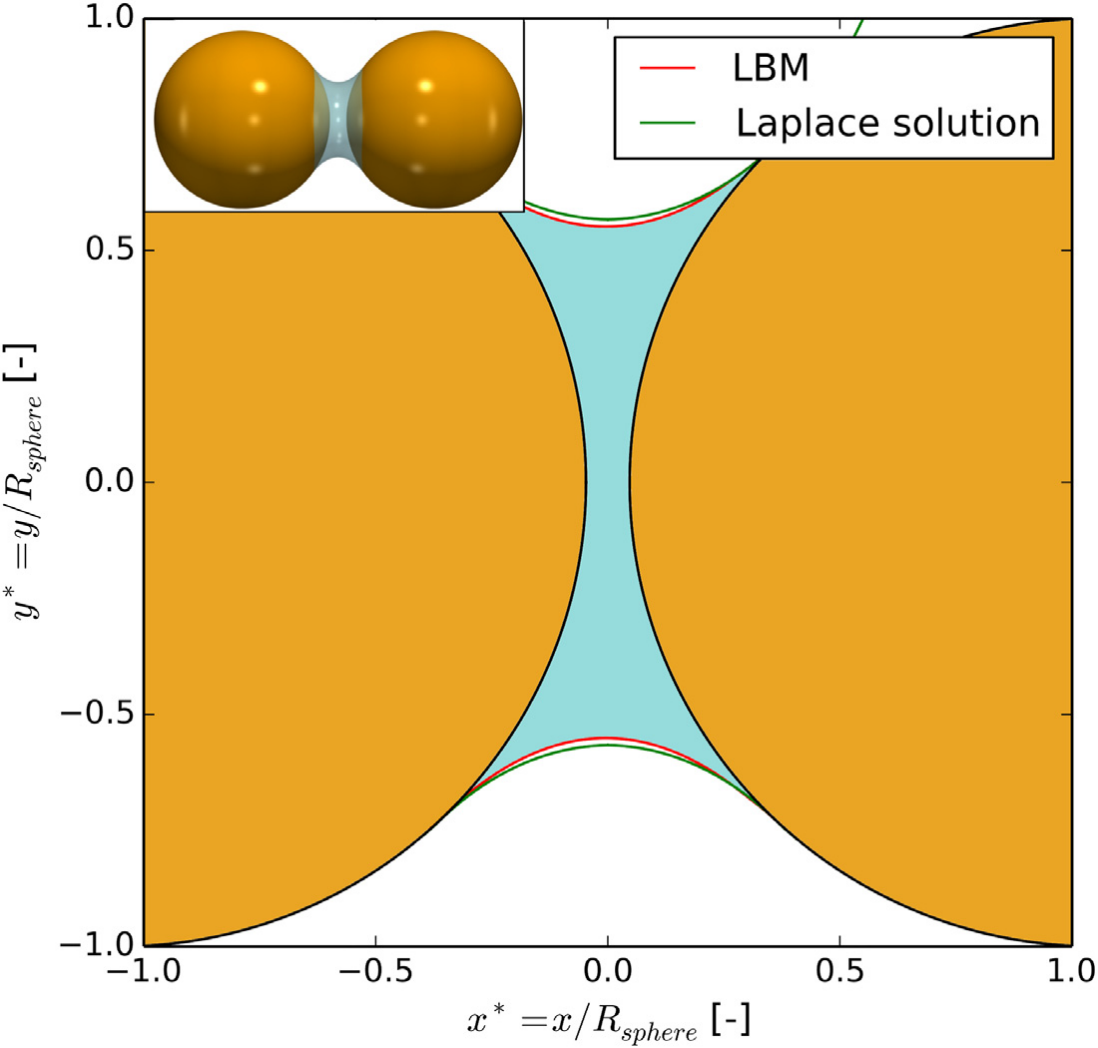
Overlapped capillary bridge profiles obtained numerically and analytically.

Fig. 14 shows the volume-pressure dependency until breakage. The LBM simulation and the Laplace–Young solution follow a very similar trend, with the relative error generally less than 10${}^{-2}$.

**Fig. 16:**
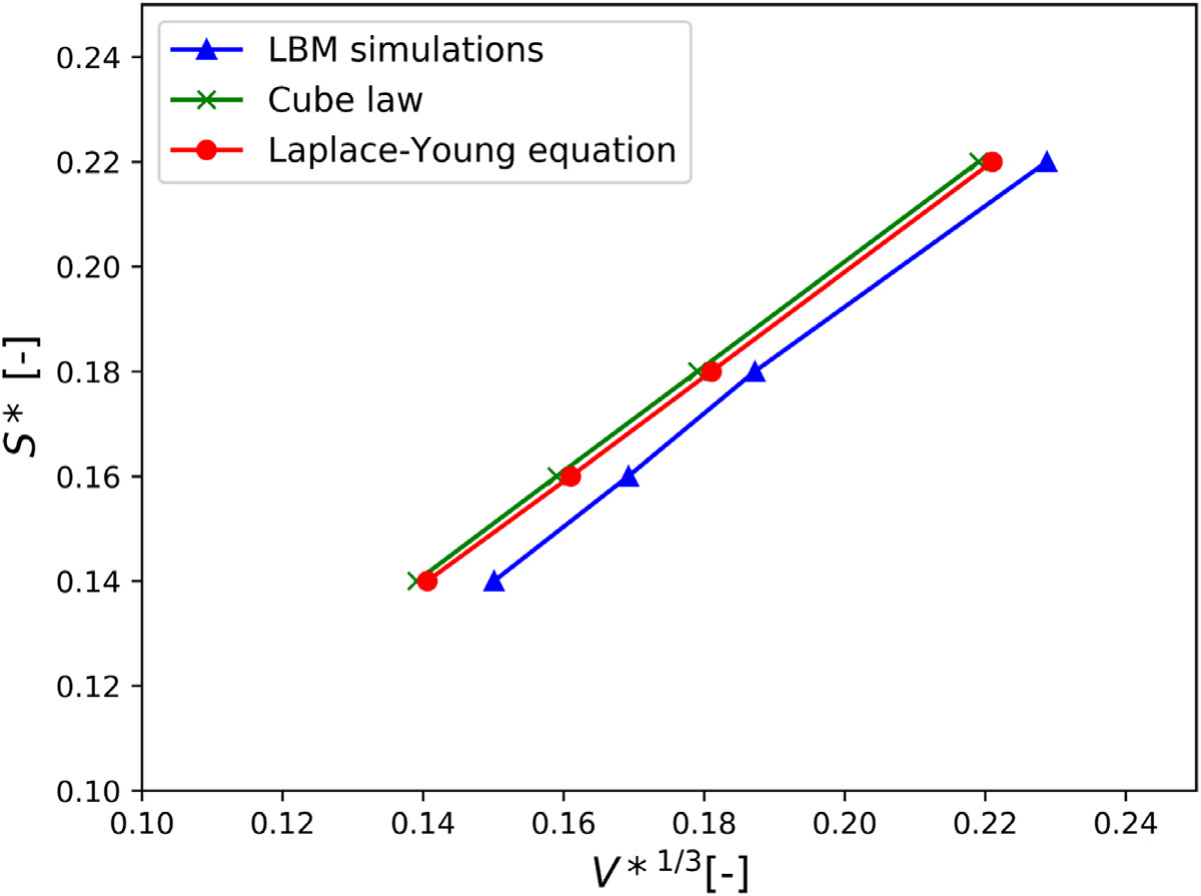
Dimensionless rupture distances ($S^{*}$) of fluid bridges between two spheres as a function of the dimensionless liquid bridge volume ($V^{*}$), calculated from Laplace-Young equation, LBM simulations and the cubic law.

Likewise, the critical distance $S_{c}$(sphere separation that leads to breakage of the bridge) can be compared. $S_{c}$can be obtained on a theoretical basis: it is the distance beyond which the Laplace-Young problem degenerates into a solutionless problem (practically approached by the upper bound of the actual solutions). Previous works [48]have shown that $S_{c}$is approximately proportional to the cubic root of the volume of the bridge. This empirical relation is also compared to the results. Fig. 16shows the rupture distance obtained by the different methods. The LBM follows a correct trend yet the distance is systematically underestimated, by 4% approximately. It is less accurate than the cubic approximation. The systematic underestimation can be explained by the difficulty to approach a mechanically unstable solution numerically.

## Conclusions

The hydrostatic properties and pore-scale morphology of immiscible phases have been obtained by the multicomponent Shan–Chen LBM for systematic comparisons with other methods. This article provides estimates of discretization errors and guidelines to calibrate the method and minimize errors.

Two-fluid-phase flow through capillary tubes has been analyzed and compared to the solution given by the MS-P method. Entry pressure, curvature and interface profile obtained from LB simulations converge to the analytical solution with mesh refinement. The capillary bridges simulated between 2 spheres also converge to the solution obtained directly from Laplace–Young equation, in terms of both shape and rupture distance. Discretization errors are introduced in part because of the solid boundaries: curved surfaces are modeled as stair-cased lines, which may not approximate the curved wall properly if the lattice resolution is not fine enough. In addition the numerical thickness of the fluid interfaces around the solids is also a source of error. These discretization errors were found to scale nearly linearly with mesh size, and relatively independently of rotations of the grid frame. For the error due to interfacial thickness we showed (section *Results*) that a significant reduction was possible with appropriate geometrical corrections of the solid boundaries. This correction leads to shrink the size of all solid objects by a mesh-dependent length to minimize the mesh-dependency of the result. This technique has been used systematically throughout this study and proved to give satisfactory results.

The aim is to progressively improve the local rules introduced in pore-network approaches from the analysis of elementary subsets, following [49]. Indeed, this article is meant to be a validation of the multicomponent Shan–Chen model to simulate multiphase flow in porous media and justify the mesh resolution and flow conditions used in [7] where an sphere packing is decomposed into a series of subsets that are solved separately using the LBM.

## Declaration of Competing Interest

The Authors confirm that there are no conflicts of interest.

## Figures and Tables

**Table A.1:** Physical and lattice units used during numerical simulations.

Quantity	Physical parameter	SI units	LB parameter
Distance between nodes	Δx	m	δx
Time step	Δt	s	δt
Velocity	v	m/s	$v_{lb}$
Density	ρ	kg/m${}^{3}$	$\rho_{lb}$
Kinematic viscosity	ν	m${}^{2}$/s	$\nu_{lb}$
Pressure	p	kg/(m s${}^{2})$	$p_{lb}$

**Fig. A1:**
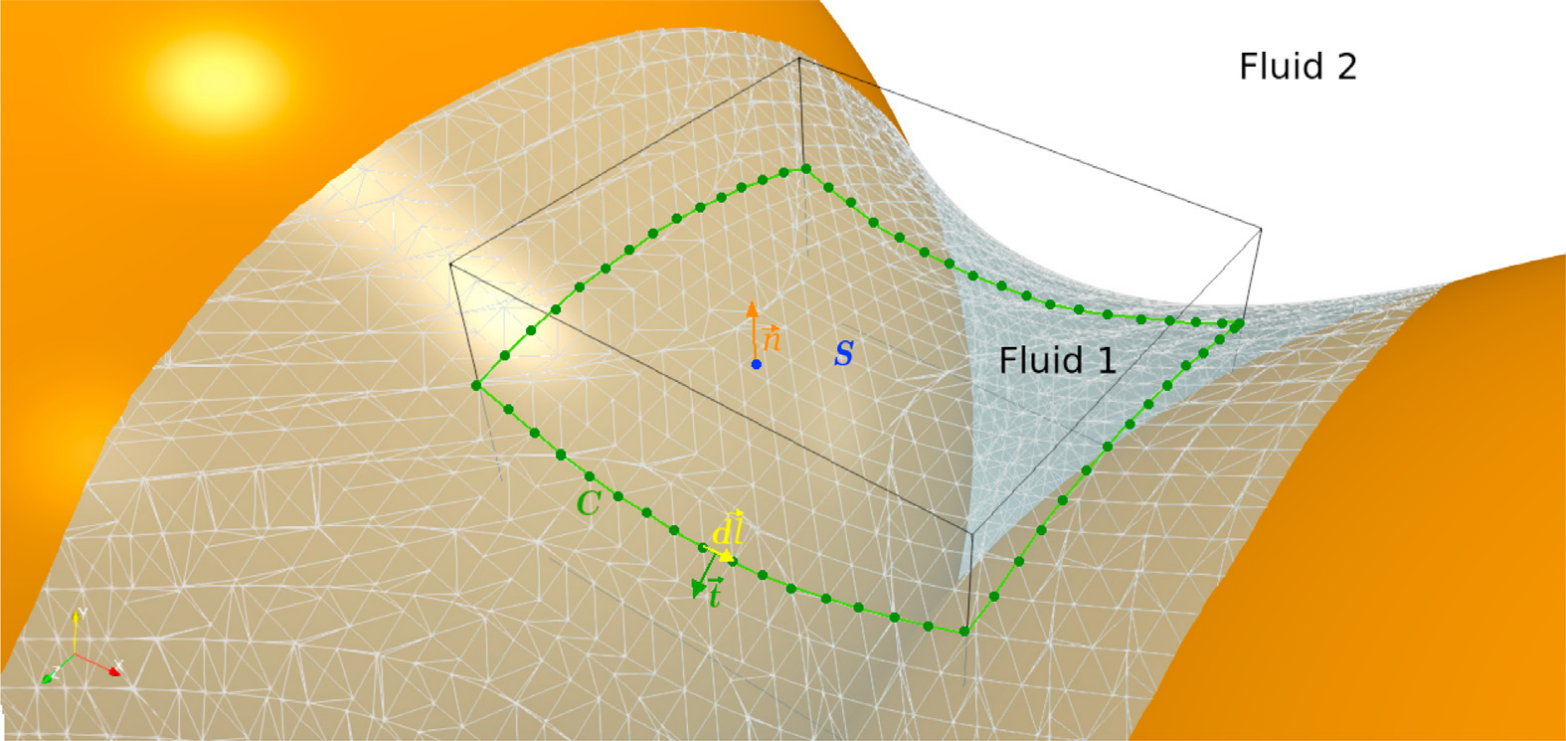
Fluid-fluid interface deformed due to surface tension. Mean curvature is determined by performing a force balance in a volume element V. Surface Sand contour Cresult from the intersection between Vand the interface. nrepresents the unit outward vector normal to S,dlthe unit vector tangent to Cand tthe unit vector normal to Cand tangent to S.

## Appendix A Appendix

### Physical and LBM units

Correlating physical properties to lattice units is an essential task in order to simulate physical problems. Moreover, choosing the right conversion will avoid stability problems and help us to have accurate results. As suggested in [50], [51], [52], physical units can be related to lattice units through unit conversion or dimensionless numbers such as the Reynold, the Froude or the Bond number. The parameters involved in the physical and the LB systems are summarized in Table A.1.

Conversion factors for length, time, velocity and density are: $C_{x}=\frac{\Delta x}{\delta x},$$C_{t}=\frac{\Delta t}{\delta t},$$C_{v}=\frac{\Delta x}{\Delta t}\frac{\delta t}{\delta x}$and $C_{\rho }=\frac{\rho }{\rho_{lb}}$. Similarly, we can find expressions for the kinematic viscosity $C_{\nu }=C_{x}^{2}/C_{t},$and pressure $C_{p}=C_{x}^{2}/C_{t}^{2}$. Kinematic viscosity is also related to the relaxation time τas

(A.1) $$\nu =c_{s}^{2}\left(\tau -\frac{1}{2}\right)\frac{\Delta x^{2}}{\Delta t}\frac{\delta t}{\delta x^{2}}$$

The method presented above is consistent and can be applied to find other quantities [51]. Nevertheless, one important constraint must be kept in mind. LBM is limited to low Mach numbers due to compressibility effects that lead to numerical instabilities [50], [53]. In order to conduct numerical simulations of quasi-compressible flows and reduce the numerical error, lattice Boltzmann velocities should be significantly smaller than the speed of sound ($v_{lb}<<c_{s}$). Dimensionless numbers are extensively used to overcome this limitation. The first step consist of converting the physical system into a dimensionless system. After that, dimensionless units are transformed into lattice units. For the sake of clarity, let us use the Bond number to illustrate the unit conversion in terms of dimensionless number. Bond number relates capillary and gravitational forces and is defined as:

(A.2) $$Bo=\frac{\rho gL^{2}}{\gamma }$$

where ρis the fluid density, gis the gravity, La characteristic length and γthe surface tension. Bomust have the same value regardless the system of units. Thus, $Bo=\frac{\rho gL^{2}}{\gamma }=\frac{\rho_{lb}g_{lb}L_{lb}^{2}}{\gamma_{lb}}$is able to correlate the lattice and physical units.

### Curvature analysis

In order to analyze the multiphase flow it is crucial to study the shape of the fluid-fluid interface. Thus, in this section we introduce a method to determine the interface curvature following [54].

Given a fluid-fluid interfacial surface Senclosed by an arbitrary volume element Vsuch as the one displayed in Fig. A.1, we can perform a force balance on V: *Inertial force = Body force + Hydrodynamic force exerted on S + Surface tension force exerted along C*, equivalently

(A.3) $$\int_{V}\rho \frac{Du}{Dt}dV=\int_{V}fdV+\int_{S}k\left(n\right)+\hat{k}\left(\hat{n}\right)dS+\int_{C}\gamma tdl$$

where,

• dlrefers to a length increment along the closed curve Cthat forms its boundary (see Fig. A.1),
• ρis the fluid density,
• γis the surface tension,
• the stress vector representing the force exerted by fluid 2 on S(see Fig. A.1) is:
  (A.4) k(n)=n·T
• the stress vector representing the force exerted by fluid 1 on Sis:
  (A.5) $$\hat{k}\left(\hat{n}\right)=\hat{n}\cdot \hat{T}=-n\cdot \hat{T}$$

The stress tensors are defined by means of the local fluid pressure and velocity gradient as

(A.6) $$T=-pI+\nu \left[\nabla u+\left(\nabla u\right){}^{T}\right]\hat{T}=-\hat{p}I+\hat{\nu }\left[\nabla \hat{u}+\left(\nabla \hat{u}\right){}^{T}\right]$$

Neglecting the acceleration and body forces leads to

(A.7) $$\int_{S}\left[k\left(n\right)+\hat{k}\left(\hat{n}\right)\right]dS+\int_{C}\gamma tdl=0$$

By assuming hydrostatic equilibrium, Eq. (21) is reduced to T=−pIand $\hat{T}=-\hat{p}I$respectively (no viscous contribution). Thus, Eq. (22) can be rewritten as:

(A.8) $$\int_{S}p_{c}ndS+\int_{C}\gamma tdl=0$$

where $p_{c}$is the pressure difference between the two fluids. Eq. (23) states that pressure jump across a static interface is balanced by the curvature at the interface, which is an integral form of the Young-Laplace equation $p_{c}=-\gamma \nabla \cdot n$(where ∇·nis the curvature), thus

(A.9) $$\gamma \int_{S}\left(-\nabla \cdot n\right)ndS+\gamma \int_{C}tdl=0,$$

and since ∇·nis constant in hydrostatic conditions

(A.10) $$\left(-\nabla \cdot n\right)\int_{S}ndS+\int_{C}tdl=0.$$

Finally, the curvature of the interface can be obtained numerically by evaluating both integrals on the basis of LBM results, given a volume element (as in Fig. A.1):

(A.11) $$\nabla \cdot n=\frac{∥\int_{C}tdl∥}{∥\int_{S}ndS∥}$$
